# 450. Performance Evaluation of PMMV as an internal control for the wastewater surveillance of SARS-CoV-2

**DOI:** 10.1093/ofid/ofad500.520

**Published:** 2023-11-27

**Authors:** Hosoon Choi, John David Coppin, Piyali Chatterjee, Emma Brackens, Lynn Mayo, Munok Hwang, Morgan Bennett, Shantini D Gamage, Chetan Jinadatha

**Affiliations:** Central Texas Veterans Health Care System, Temple, Texas; Central Texas Veterans Health Care System, Temple, Texas; Central Texas Veterans Health Care System, Temple, Texas; Central Texas Veterans Health Care System, Temple, Texas; Central Texas Veterans Health Care System, Temple, Texas; Central Texas Veterans Health Care System, Temple, Texas; Central Texas Veterans Health Care System, Temple, Texas; National Infectious Diseases Service, Cincinnati, Ohio; Central Texas Veterans Health Care System, Temple, Texas

## Abstract

**Background:**

SARS-CoV-2 RNA is shed into sewers by infected individuals via excreta and sputum. Detections of SARS-CoV-2 RNA in wastewater can correlate with local COVID-19 prevalence. Wastewater-based surveillance can be a valuable tool for supplementing clinical testing in controlling COVID-19 transmission. Pepper mild mottle virus (PMMV) RNA has been used as a normalization biomarker in wastewater, largely reported for community-level surveillance. Here, we report the abundance of PMMV in wastewater collected during a pilot project of building-level wastewater-based SARS-CoV-2 surveillance and we evaluated the use of PMMV RNA as an internal control.

**Methods:**

Wastewater samples were collected (January 11, 2021, to July 2, 2021) at eight nursing homes located across the US and shipped overnight for processing. After the heat inactivation by incubating samples in a 65±1°C circulating water bath for 90 minutes, the virus in the wastewater was concentrated using InnovaPrep Concentrating Pipette Select, and RNA was isolated from the concentrate and subjected to reverse transcription quantitative PCR (RT-qPCR) and RT-digital PCR to detect SARS-CoV-2 and PMMV RNA.

Steps of building-level wastewater-based SARS-CoV-2 surveillance

**Figure:**
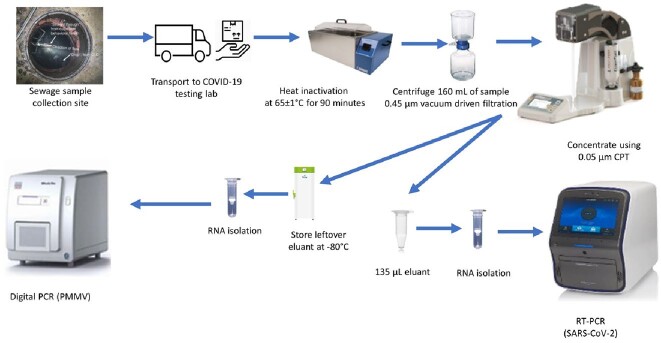

**Results:**

Mean copy number and level of variability of PMMV in wastewater were different among the nursing home sites (Figure 1). SARS-CoV-2 was detected in sites with both low and high mean PMMV and variability (Figure 1). The distribution of PMMV for negative, inconclusive, and positives were not different (Figure 2) and no correlation between RNA concertation of SARS-CoV-2 and PMMV in the wastewater was detected (Figure 3).

**Figure 1.**
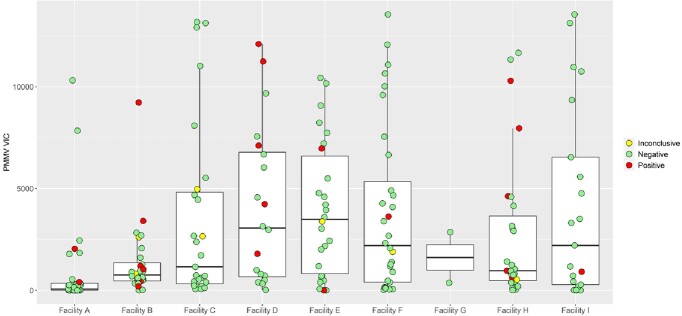
Concentration of PMMV RNA in wastewater of each collection site.

**Figure 2.**
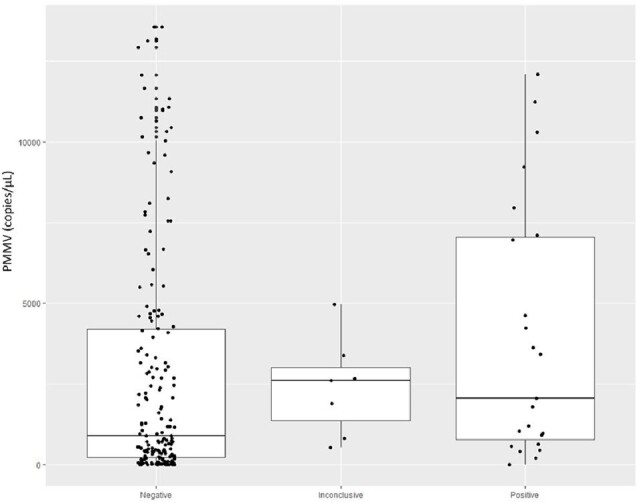
Concentration of PMMV RNA in wastewater samples with negative, inconclusive, or positive SARS-CoV-2 detection.

**Figure 3.**
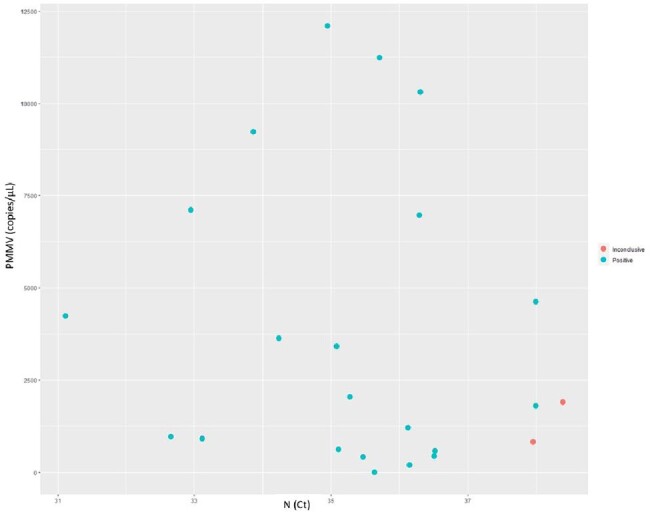
Correlation between PMMV RNA and SARS-CoV-2 RNA in wastewater samples.

**Conclusion:**

PMMV RNA has been used to control noise due to varying human waste input. The role of PMMV as an internal control was very limited for this building-level surveillance pilot project. This could be mainly due to very low SARS-CoV-2 viral load in the wastewater samples. In this wastewater surveillance project, the variation of PMMV RNA was too big to be used as normalization factor. Also, the clear cutoff level of PMMV for SARS-CoV-2 detection in the wastewater could not be defined. More controlled studies are needed to determine the usability of PMMV as normalization biomarkers in building level-wastewater SARS-CoV-2 surveillance.

**Disclosures:**

**All Authors**: No reported disclosures

